# Characterization and phylogenetic analysis of the complete chloroplast genome of *Ilex rotunda*, traditional Chinese medicine plant

**DOI:** 10.1080/23802359.2021.1967804

**Published:** 2021-08-31

**Authors:** Hong Chen, Xinran Chong, Yanwei Zhou, Linhe Sun, Fan Zhang, Xiaoqing Lu, Naiwei Li, Ting Zhou, Yunlong Li

**Affiliations:** Jiangsu Key Laboratory for the Research and Utilization of Plant Resources, Institute of Botany, Jiangsu Province and Chinese Academy of Sciences, Nanjing, China

**Keywords:** Aquifoliaceae, *Ilex rotunda*, chloroplast genome, phylogenetic analysis

## Abstract

*Ilex rotunda* is a traditional Chinese medicine plant. In this study, we characterized the complete chloroplast (cp) genome sequence of *I. rotunda* to investigate its phylogenetic relationship. The cp genome of *I. rotunda* was 157,743 bp in length with 37.62% overall GC content, including a large single-copy (LSC) region of 87,060bp, a small single-copy (SSC) region of 18,432 bp, which were separated by a pair of inverted repeats (IRS) of 26,121 bp. The cp genome contained 133 genes, including 88 protein-coding genes, 37 tRNA genes, and 8 rRNA genes. Phylogenetic analysis based on whole cp genome sequences showed that *I. rotunda* is closely related to *I. pubescens* and *I. polyneura*.

## Introduction

*Ilex rotunda* Thunberg 1784 belongs to *Ilex* Linnaeus which is within the monogeneric family, i.e. Aquifoliaceae, that includes ca. 700 species (Yao et al. 2021). *Ilex* have two diversity centers, South East Asia-Malesia and South America, but few species in Europe and Africa. China is one of the *Ilex* diversity centers that there are 200 species recognized (Xie et al. 2019). In China, more than 40 species and their varieties, including *I. rotunda*, are recorded for the medicinal purpose (Hao et al. 2015). In terms of medicinal value, *Ilex* is effective to clearing away heat and detoxification, detumescence and pain relief. Accordingly, China is one of the major countries that have abundant natural *Ilex* resources. However, the morphological study based on leaves, inflorescence, and fruit is difficult to distinguish close relative species within *Ilex*. The molecular phylogeny methods can be a useful tool for systematics study. The complete chloroplast genome can provide a framework to insight *Ilex* phylogenetic relationship (Nei and Kumar 2000). *Ilex rotunda* is widely distributed is southern China, that can be used for traditional Chinese medicine and for garden plant.

*I. rotunda* was planted in Nanjing Botanical Garden, Mem. Sun Yat-sen (118°49′55″E, 32°3′32″N), Nanjing, China. The voucher specimen (NO.NBGJIB-Ilex-0056) was deposited in the Institute of Botany, Jiangsu Province and Chinese Academy of Science (http://www.cnbg.net/, hongchen, chenhong@cnbg.net). Approximately 3 g of fresh leaves were collected and stored in liquid nitrogen for chloroplast DNA isolation. The qualified DNA was used for library construction and sequencing (Illumina, San Diego, CA). In total, 6710.3 Mb of raw data and 6352.4 Mb clean data were obtained. *De novo* genome assembly and annotation were conducted by the organelle assembler NOVOPlasty (Dierckxsens et al. 2017) and GeSeq (Tillich et al. 2017), respectively. The complete cp genome was deposited in GenBank (accession number: MW292559).

The shape of the cp genome of *I. rotunda* was a double-stranded closed loop. The cp genome of *I. rotunda* was 157,743 bp in length, including a large single-copy (LSC) region of 87,060 bp, a small single-copy (SSC) region of 18,432 bp. The cp genome contained 133 genes, including 88 protein-coding genes, 37 tRNA genes, and 8 rRNA genes. For the 132 identified genes, 14 contained introns, 4 (*rps12*, *ycf3*, *rps12*, *clpP*) had two introns and the rests had a single intron. The overall GC content of the circular genome was 37.62%.

**Figure 1.**
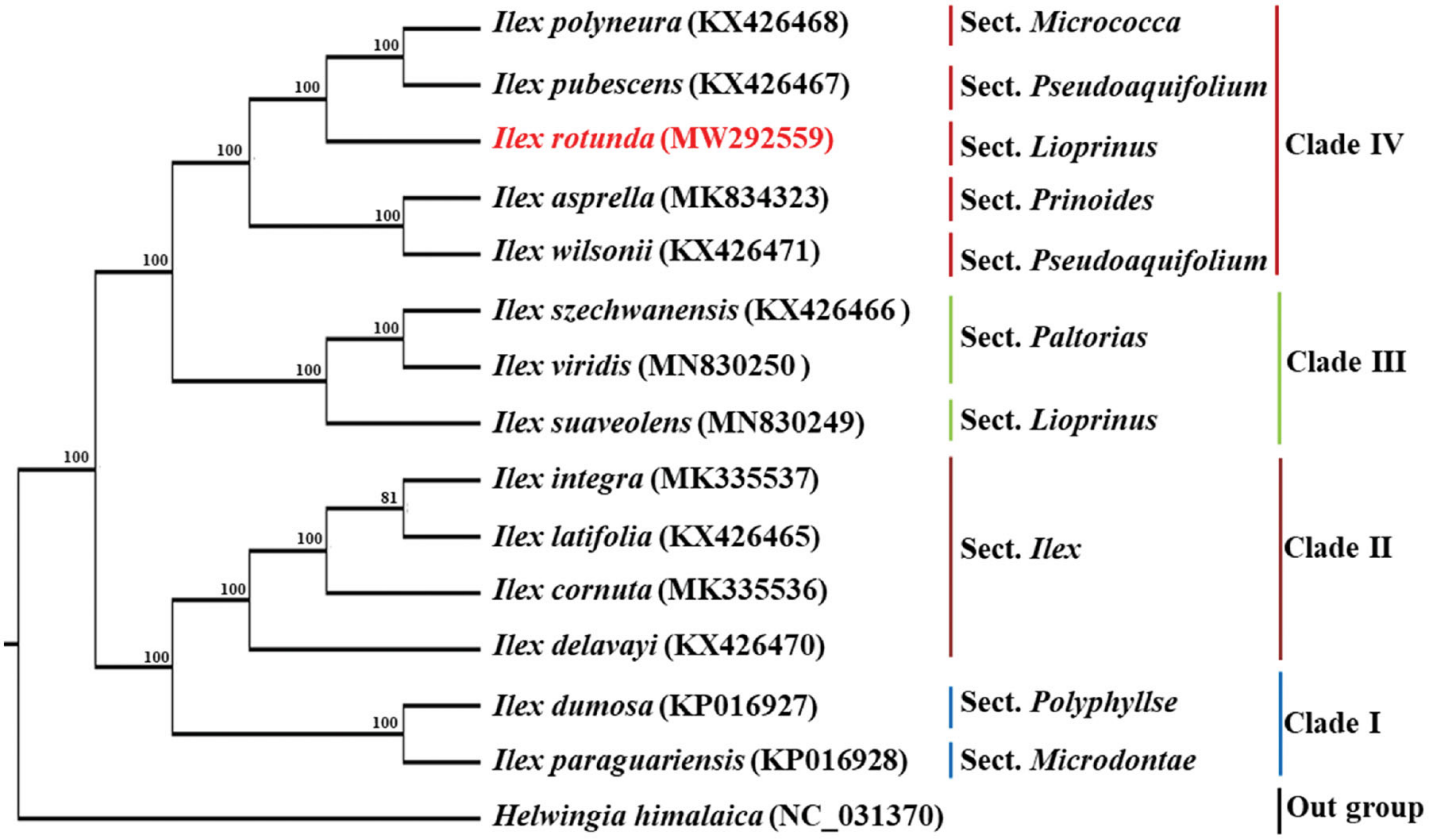
Phylogenetic tree based on the cp genome sequences of Ilex rotunda and 13 other species (contain 1 outgroup). Section names were displayed in the right side of phylogenetic tree (Su et al. 2020). Numbers on the nodes indicate bootstrap values.

To explore the evolution status of *Ilex rotunda*, 14 other complete chloroplast genome sequences were downloaded from the Genebank database (*Helwingia himalaica* as outgroup). Sequences alignment was firstly performed by MAFFT (Katoh and Standley 2013), and then phylogenetic tree was constructed with maximum-likelihood method, using the IQ-tree program (Nguyen et al. 2015). Our phylogenetic analysis indicated that all the examined *Ilex* species were divided into four clades, and *I. rotunda* has a close relationship with *I. pubescens* and *I. polyneura* (Figure 1). The work herein provided a better understanding of *I. rotunda,* and plays a critical role in constructing phylogeny of Aquifoliaceae.

## Data Availability

The genome sequence data that obtained at this study are openly available in GenBank of NCBI (https://www.ncbi.nlm.nih.gov/) under accession number of MW292559. The associated BioProject, Bio-Sample and SRA, numbers are PRJNA747869, SAMN20298920 and SRR15195658, respectively.
